# Следы отбора и гены-кандидаты адаптации к экстремальным факторам среды в геномах турано-монгольских пород крупного рогатого скота

**DOI:** 10.18699/VJ21.023

**Published:** 2021-03

**Authors:** N.S. Yudin, A.A. Yurchenko, D.M. Larkin

**Affiliations:** Institute of Cytology and Genetics of the Siberian Branch of the Russian Academy of Sciences, Novosibirsk, Russia; Institute of Cytology and Genetics of the Siberian Branch of the Russian Academy of Sciences, Novosibirsk, Russia; Institute of Cytology and Genetics of the Siberian Branch of the Russian Academy of Sciences, Novosibirsk, Russia The Royal Veterinary College, University of London, London, United Kingdom

**Keywords:** cattle, Bos taurus, Bos indicus, Turano-Mongolian cattle, adaptation, genome, selection signatures, cold, immunity, highlands, крупный рогатый скот, Bos taurus, Bos indicus, турано-монгольский скот, адаптация, геном, следы отбора, холод, иммунитет, высокогорье.

## Abstract

Изменения, происходящие в окружающей среде, заставляют популяции организмов адаптироваться к новым условиям либо за счет фенотипической пластичности, либо за счет генетических или эпигенетических изменений. Следы отбора, такие как специфические изменения частот аллелей и гаплотипов, снижение или повышение генетического разнообразия, помогают выявить изменения генома крупного рогатого скота в ответ на искусственный и естественный отбор, а также локусы и варианты, непосредственно влияющие на адаптивные и экономически важные признаки. Достижения генетики и биотехнологии дают возможность быстрого переноса уникальных генетических вариантов, возникших у местных пород крупного рогатого скота в процессе адаптации к локальной среде обитания, в геномы интернациональных высокопроизводительных пород с целью сохранения их выдающихся свойств в новых условиях обитания. Возможно и использование методов геномной селекции для повышения частот адаптивных аллелей у интернациональных пород. В обзоре рассмотрены недавние работы по истории происхождения и эволюции турано-монгольских пород крупного рогатого скота, адаптации турано-монгольского скота к экстремальным условиям среды. Сделано обобщение имеющихся сведений о потенциальных генах-кандидатах адаптации в геномах турано-монгольских пород, включая гены устойчивости к холоду, гены иммунного ответа и гены адаптации к высокогорью. Авторы приходят к выводу, что имеющиеся данные литературы не позволяют отдать предпочтение одному из двух возможных сценариев происхождения турано-монгольских пород – в результате доместикации дикого тура на территории Восточной Азии или вследствие миграции тауринной протопопуляции из Ближнего Востока. Турано-монгольским породам свойственна высокая адаптация к экстремальным климатическим условиям (холод, жара и недостаток кислорода в горах) и паразитам (гнус, клещи, бактериальные и вирусные инфекции). В результате высокопроизводительного генотипирования и секвенирования геномов и транскриптомов представителей этих пород в последнее время были выявлены перспективные гены-кандидаты и генетические варианты, участвующие в адаптации к факторам внешней среды.

## Abstract (en)

Changes in the environment force populations of organisms to adapt to new conditions, either through phenotypic plasticity or through genetic or epigenetic changes. Signatures of selection, such as specific changes in the frequency of alleles and haplotypes, as well as the reduction or increase in genetic diversity, help to identify changes in the cattle genome in response to natural and artificial selection, as well as loci and genetic variants directly affecting adaptive and economically important traits. Advances in genetics and biotechnology enable a rapid transfer of unique genetic variants that have originated in local cattle breeds in the process of adaptation to local environments into the genomes of cosmopolitan high-performance breeds, in order to preserve their outstanding performance in new environments. It is also possible to use genomic selection approach to increase the frequency of already present adaptive alleles in cosmopolitan breeds. The review examines recent work on the origin and evolution of Turano-Mongolian cattle breeds, adaptation of Turano-Mongolian cattle to extreme environments, and summarizes available information on potential candidate genes for climate adaptation of Turano-Mongolian breeds, including cold resistance genes, immune response genes, and high-altitude adaptation genes. The authors conclude that the current literature data do not provide preference to one of the two possible scenarios of Turano-Mongolian breed origins: as a result of the domestication of a wild aurochs at East Asia or as a result of the migration of taurine proto-population from the Middle East. Turano-Mongolian breeds show a high degree of adaptation to extreme climatic conditions (cold, heat, lack of oxygen in the highlands) and parasites (mosquitoes, ticks, bacterial and viral infections). As a result of high-density genotyping and sequencing of genomes and transcriptomes, prospective candidate genes and genetic variants involved in adaptation to environmental factors have recently been identified

## Введение

Локальные породы крупного рогатого скота (КРС) могут обладать ценными генетическими вариантами для проведения селекции и создания новых пород в ответ на возникающие вызовы животноводству, включая изменение климата, появившиеся или возрождающиеся угрозы заболеваний, инновации в области диетологии и изменившиеся запросы рынка (Kantanen et al., 2015). Достижения генетики и биотехнологии дают возможность быстрого переноса уникальных генетических вариантов, возникших у местных пород КРС в процессе адаптации к локальной среде обитания, в геномы коммерческих интернациональных высокопроизводительных пород с целью сохранения их выдающихся свойств в новых условиях обитания (Stranden et al., 2019). Для повышения частот адаптивных аллелей у коммерческих пород возможно и использование методов геномной селекции. Однако для успешного применения этих технологий недостаточно знать гены, вовлеченные в адаптацию или устойчивость к болезням; надо знать, какие именно генетические варианты вносят вклад в требуемый признак. То есть необходимо изучать геномы КРС на нуклеотидном уровне и при этом учитывать исторические взаимоотношения между породами и условия их формирования для того, чтобы отличить функционально важные генетические варианты от результатов генетического дрейфа и прохождения «бутылочного горлышка».

Целью настоящего обзора являлся анализ современного состояния проблемы происхождения и эволюции турано-монгольских породКРС, а также обобщение имеющихся сведений о потенциальных генах-кандидатах, которые вносят вклад в адаптацию этих пород к экстремальным условиям внешней среды.

## Методы

Поиск и отбор литературы были выполнены в соответствии с общепринятыми критериями, предъявляемыми к систематическим обзорам (Pautasso, 2013). Предварительно нами был составлен список турано-монгольских пород КРС (Приложение 1)^1^ (Porter et al., 2016; Лазебная и др., 2018). Далее мы провели систематический поиск литературы, опубликованной в базах данных PubMed, Scopus, Web of Science и Google Scholar с января 2010 г. (начало широкого применения методов массового параллельного секвенирования) до января 2020 г. с использованием следующих поисковых запросов: «[Название породы] AND Cattle AND Whole genome genotyping», «[Название породы] AND Cattle AND Whole genome sequencing», «[Название породы] AND Cattle AND Transcriptome sequencing», «[Название породы] AND Cattle AND Selection signatures». Критериями для включения публикации в обзор служили: исследование хотя бы одной турано-монгольской породы либо ее гибрида с другими породами; описание секвенирования, полногеномного генотипирования или транскриптома генома турано-монгольской породы; результаты поиска следов позитивного отбора в геноме либо идентификация полиморфизма по числу копий ДНК (CNV).

Приложения 1 и 2 см. по адресу: http://www.bionet.nsc.ru/vogis/download/pict-2021-25/appx6.pdf


**Адаптация турано-монгольских пород к экстремальным условиям среды**

Турано-монгольский скот – группа пород КРС, которые разводят преимущественно в Азии (см. Прил. 1) (Моисеева и др., 2006; Porter et al., 2016). По морфологии тураномонгольский скот отличается от европейских тауринных пород, особенно по форме черепа и рогов (Felius et al., 2011). Череп имеет клиновидную форму, узкую корону и углубление на лобной кости. Рога направлены вверх, а не вперед, как у большинства тауринных пород.

Многие породы турано-монгольского КРС проявляют большую выносливость и устойчивость к отрицательным температурам в результате адаптации к суровому азиатскому климату. В частности, породы азиатских степей способны выдерживать годовые колебания температуры от –50 до +35 °С (Моисеева и др., 2006). Особую адаптацию демонстрирует самая северная порода КРС в мире – якутский скот, центр разведения которого находится вблизи Северного полюса холода. Ряд морфологических признаков, таких как толстая зимняя шерсть, маленькие, покрытые мехом вымя и мошонка, эффективная терморегуляция и замедленный метаболизм при низких температурах, приводят к чрезвычайной устойчивости якутского скота к экстремальному холоду (Dmitriev, Ernst, 1989; Tapio et al., 2010). Для турано-монгольских пород характерны физическая выносливость, минимальное участие человека в их содержании, круглогодичное пребывание на свободном выпасе и сохранение жизнеспособности при низкокалорийной и скудной кормовой базе в отдельные периоды года, устойчивость к гнусу, способность переваривать грубые корма и находить их под снежным покровом (Лазебная и др., 2018). Также якутский скот, вероятно, обладает устойчивостью к туберкулезу, лейкозу и бруцеллезу (Dmitriev, Ernst, 1989).

Китайский турано-монгольский скот издавна использовался в качестве тягловой силы и ценится за свою устойчивость к паразитам, толерантность к факторам окружающей среды и физическую выносливость (Huai et al., 1993). Считается, что южный китайский скот устойчив к сырости, жаре и клещам. Северный скот, с более толстой кожей и густым волосяным покровом, устойчив к холоду и клещам. Кроме того, высокогорная тибетская порода КРС хорошо адаптирована к холоду и недостатку кислорода в высокогорных районах (Wu D.D. et al., 2018). По сравнению с чистым тауринным скотом, например голштинским, южно-китайская смешанная индицинно-тауринная порода Yunnan humped имеет высокую резистентность к тейлериозу, туберкулезу и клещам (Chen Y. et al., 2019).

**Происхождение и эволюция турано-монгольских пород**

Генетическая обособленность некоторых турано-монгольских пород КРС, включая якутскую, калмыцкую и монгольскую, была показана с использованием полилокусных ISSR-маркеров (Генджиева, Сулимова, 2012), а также микросателлитных ДНК-маркеров (Li M.H. et al., 2007; Svishcheva et al., 2020). Например, якутская порода выделялась в отдельный кластер при анализе 48 европейских пород по 19 микросателлитным маркерам (Li M.H., Kantanen, 2010). Другими авторами также была выявлена высокая обособленность якутского скота по сравнению с девятью другими породами КРС по данным полногеномного генотипирования. При этом калмыцкая порода оказалась родственной сербской породе буша (Iso-Touru et al., 2016), что соответствует полученным ранее этой группой данным по генетическому разнообразию мтДНК, Y-хромосомы и микросателлитных ДНК-маркеров у этих пород (Kantanen et al., 2009; Li M.H., Kantanen, 2010). Согласно результатам полногеномного анализа однонуклеотидных полиморфизмов (ОНП) девяти российских пород и 45 пород Евразии, якутская и калмыцкая породы формировали отдельный отдаленный кластер на дендрограмме только российских пород, построенной по алгоритму «сеть соседей» (Neighbor-Net) (Sermyagin et al., 2018), а на дендрограмме всех евразийских пород входили в кластер пород турано-монгольского корня.

Вполне вероятно, что многие турано-монгольские породы в доисторические и исторические времена скрещивались с зебу (Bos indicus) (Peilieu, 1984; Huai et al., 1993; Kantanen et al., 2009), бантенгом (Bos javanicus) (Chen N. et al., 2018b; Zhang W. et al., 2018) и яком (Bos grunniens) (Xia et al., 2019). Исследования структуры популяций турано-монгольского скота в Китае по мтДНК показывают, что гаплогруппы Bos taurus более распространены в Cеверном Китае, гаплогруппы Bos indicus – в Южном, а породы КРС в Центральном Китае демонстрируют промежуточные частоты мтДНК зебу (Lai et al., 2006). При изучении ДНК Y-хромосомы в популяциях китайского КРС получена аналогичная географическая картина: гаплогруппа Bos taurus Y2 преобладает на севере, а гаплогруппа Bos indicus Y3 – на юге Китая (Lu et al., 2017). В настоящее время многие турано-монгольские породы практически исчезли, другие для повышения продуктивности были разбавлены вливанием крови импортных тауринных пород и часто находятся под угрозой дальнейшей гибридизации (Peilieu, 1984; Huai et al., 1993; Kantanen et al., 2009; Gotoh et al., 2018). И только несколько турано-монгольских пород разводятся «в чистоте», например, в 2010 г. численность якутского скота составляла около 1200 животных (Tapio et al., 2010).

Чтобы оценить степень генетического родства и чистопородность современных турано-монгольских пород, мы собрали коллекцию опубликованных генотипов ОНП различных пород скота (Iso-Touru et al., 2016; Gao et al., 2017; Yurchenko et al., 2018b; Zhang Y. et al., 2020), насчитывающую 2676животных из 198 пород (включая 513животных из 23 турано-монгольских пород, Приложение 2).

После объединения и фильтрации всех файлов с генотипической информацией мы получили 18 250 высококачественных ОНП для дальнейших исследований. С использованием программы rapidNJ для индивидуальных особей (Simonsen et al., 2010) была построена дендрограмма на основе метода ближайшего соседства. Большинство животных группировалось в соответствии со своими породами, формируя отдельные кластеры, относящиеся к Bos indicus, Bos taurus и африканским тауринам (см. рисунок, а). Породы турано-монгольского происхождения компактно группировались в стволе тауриновой клады, что, вероятно, отражает их древнее общее происхождение. Кластеризация животных методом ADMIXTURE (Alexander, Lange, 2011) позволила выявить при четырех-пяти кластерах отдельную предковую компоненту, выделяющую турано-монгольские породы (см. рисунок, б, фиолетовый цвет). Эта компонента почти полностью определяла якутскую породу и присутствовала в значительном количестве в остальных турано-монгольских животных (см. рисунок, в), особенно в китайских, японских и корейских породах. Ближайшей к якутской по фракции турано-монгольского скота (>90 %) породой оказалась японская порода мишима, которая никогда не смешивалась с европейскими тауринами и разводится изолированно в чистоте на острове Мишима в Японии (см. Прил. 1). В остальных породах фракция турано-монгольского скота была ниже 75 %. Таким образом, несмотря на активное смешение с коммерческими породами, древний предковый генетический компонент турано-монгольского скота все еще присутствует во многих разводимых в Азии породах.

**Fig. 1.**
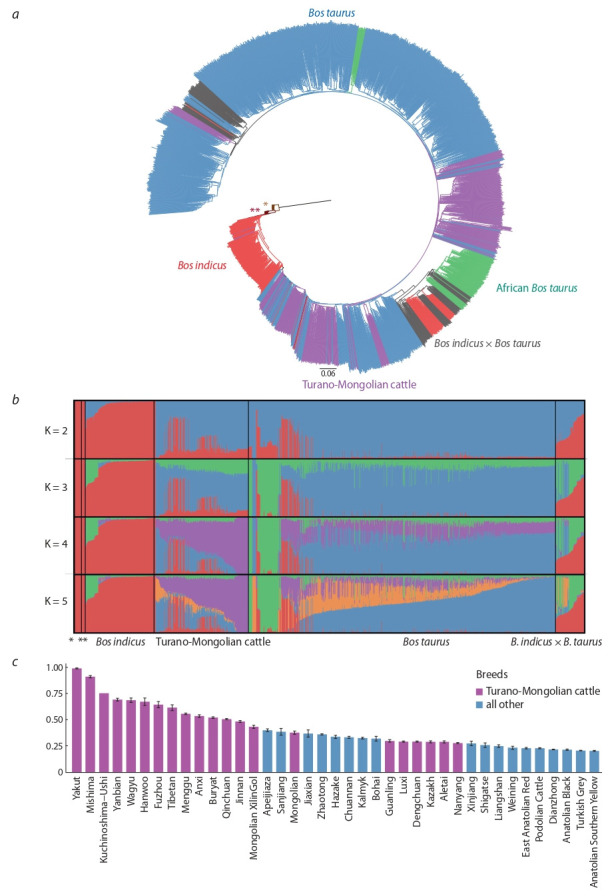
Thoroughbredness and genetic relations of modern Turano-Mongolian breeds a – Dendrogram based on 18,250 single-nucleotide polymorphisms (SNPs), built by the neighbour-joining method. The dendrogram is rooted by the yak (Bos grunniens (*)) and the banteng (Bos javanicus (**)). Purple color marks Turano-Mongolian cattle; blue, taurine breeds; red, indicine cattle; green, African breeds; gray, taurine-indicine hybrid cattle. b – Clustering of samples by ADMIXTURE for K=2 to 5 based on ancestral allele frequencies. c – The proportion of the purple (Turano-Mongolian) generics for K = 5 (by the ADMIXTURE method) in different cattle breeds.

Большинство исследователей считают, что КРС произошел от вымершего дикого тура (Bos primigenius) в ходе двух независимых событий доместикации: одно из них случилось на Ближнем Востоке около 8000–10 000 лет до н. э. и привело к возникновению безгорбого тауринного скота (Bos taurus), другое – в Южной Азии примерно в 6000–8000 лет до н. э., в результате чего появился горбатый индицинный скот зебу (Bos indicus) (Bradley, Magee, 2006; Bollongino et al., 2012). Однако Ларсон и Бюргер (Larson, Burger, 2013) отмечают, что характер ветвления филогенетических деревьев мтДНК и Y-хромосомной ДНК, на котором основана гипотеза о существовании двух центров доместикации, тоже мог сформироваться в результате целого ряда сценариев, включая географическую изоляцию популяций, генетический дрейф или гибридизацию. По мнению этих авторов, необходимы дополнительные генетические сведения, прежде чем мы сможем исключить возможность того, что индицинный скот произошел в результате гибридизации предков тауринного скота с морфологически различающимися популяциями диких туров в Южной Азии.

Текущие данные по мтДНК свидетельствуют о том, что европейский домашний скот произошел от ближневосточных разновидностей Bos taurus (Bradley, Magee, 2006). Происхождение африканских Bos taurus все еще обсуждается (Bradley, Magee, 2006), но тесная связь гаплогруппы T1 с гаплогруппами T2 и T3 позволяет думать, что она также возникла на Ближнем Востоке (Achilli et al., 2008). Ранее было установлено, что геном якутской породы содержит гаплогруппу Т, а именно ее уникальный вариант Т4, который характерен только для турано-монгольских пород (Kantanen et al., 2009). Наличие Т4 митохондриальной гаплогруппы у вагу, ханву (Mannen et al., 2004) и якутского скота (Kantanen et al., 2009) позволило предположить, что все турано-монгольские породы могут являться потомками тауринного скота, независимо доместицированного в Азии (Mannen et al., 2004; Lai et al., 2006). Однако более поздние исследования показывают, что гаплогруппа T4, вероятно, произошла от гаплогруппы T3 (Achilli et al., 2009).

Недавно нами было проведено полногеномное генотипирование представителей 18 пород КРС, разводимых в России, и осуществлено их сравнение с ранее генотипированными 135 мировыми породами КРС (Yurchenko et al., 2018b). Полученные результаты анализа филогении и общих гаплотипов выявили близкое родство бурятской и особенно якутской пород с другими азиатскими тураномонгольскими породами (вагу, ханву, монгольский скот), что может означать их раннее отделение от остальной части тауринного генофонда и, возможно, независимую доместикацию в Азии. Zhang H. с коллегами (Zhang H. et al., 2013), проанализировав найденную в Северо-Восточном Китае в районе Харбина нижнюю челюсть КРС, датируемую 10 600 годами до н. э., предположили, что древние люди могли экспериментировать с содержанием диких животных в неволе. Сравнение между мтДНК из нижней челюсти и митогеномами современных видов КРС показало, что древняя мтДНК принадлежит к новой и уникальной гаплогруппе типа С. Тем не менее ряд авторов считает, что такие утверждения преждевременны (Lu et al., 2017). По их мнению, найденная челюсть принадлежит ныне вымершей восточноазиатской форме Bos primigenius, которая не внесла генетического вклада в возникший позднее домашний скот

Таким образом, существуют два возможных сценария доместикации турано-монгольского скота: 1) домашний скот был независимо одомашнен коренным населением в Восточной Азии от диких туров (Bos primigenius); 2) тауринный домашний скот был одомашнен на Ближнем Востоке и затем завезен в Восточную Азию, а наблюдаемые различия являются результатом локальной адаптации и/или гибридизации. Существующих зооархеологических и генетических данных недостаточно, чтобы сделать однозначный вывод о доместикации турано-монгольских пород на территории современного Китая.

## Гены-кандидаты адаптации в геномах турано-монгольских пород

Далее мы сосредоточимся на анализе потенциальных генов-кандидатов, участвующих в формировании генетической адаптации к экстремальным условиям среды у турано-монгольских пород КРС, в том числе по данным полногеномного секвенирования (см. таблицу). По сравнению с данными полногеномного генотипирования ОНП, результаты полногеномного секвенирования дают бόльшую статистическую мощность при выявлении следов отбора и лучшее разрешение для локализации потенциальных генов-кандидатов и генетических вариантов, непосредственно влияющих на адаптацию, поскольку они не привязаны к аллелям, размещенным на чипах, которые нередко представлены только в популяциях, использованных для создания чипа (Boitard et al., 2016).

**Table 1.**
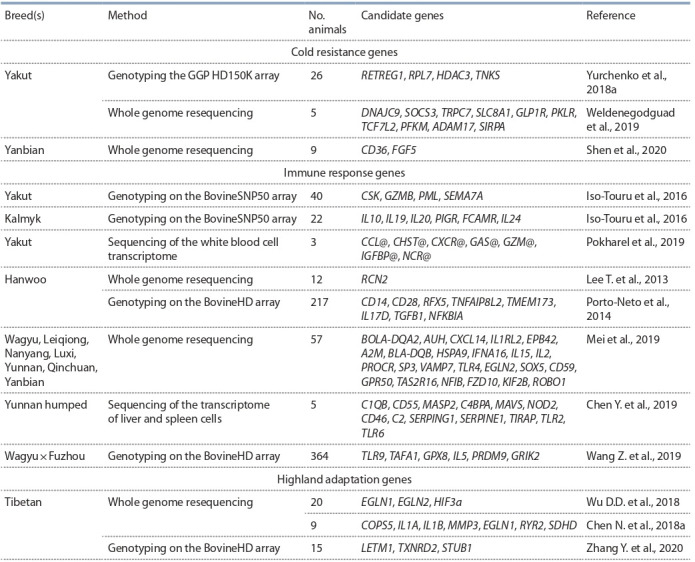
Candidate adaptation genes in the genomes of Turano-Mongolian cattle breeds

**Гены устойчивости к холоду**

Полногеномное генотипирование (более 100 тысяч ОНП) позволило выявить уникальные следы отбора в геноме якутской породы, которые могут быть непосредственно связаны с адаптацией к холодному климату. Представляет интерес обнаружение следов селекции в районе гена RETREG1 (Yurchenko, 2018а). У человека белок RETREG1 участвует в восприятии нейронами сигналов боли и холо-да (Islam et al., 2018). Мутации в этом гене у людей вызывают наследственную невропатию, сопровождающуюся неспособностью ощущать боль и температуру окружающей среды (Kurth et al., 2009). Рибосомальный ген RPL7 в районе селекции у якутской породы показывает различную экспрессию у устойчивых и чувствительных к замораживанию лягушек (Wu S. et al., 2008). Следы селекции были зафиксированы у якутского скота также в районе гена HDAC3, белок которого стимулирует термогенез в бурой жировой ткани путем активации энхансеров, и гена TNKS, участвующего в энергообмене и формировании жировой ткани у мышей (Yeh et al., 2009).

Данные полногеномного секвенирования животных якутской породы позволили выявить в ее геноме 1442 гена, которые содержали более пяти несинонимичных ОНП (Weldenegodguad et al., 2019). Были найдены следы отбора в ряде генов (DNAJC9, SOCS3, TRPC7, SLC8A1, GLP1R, PKLR и TCF7L2), которые, вероятно, связаны с адаптацией к холоду у коренного населения Сибири (Cardona et al., 2014). Причем ген SLC8A1, белковый продукт которого участвует в ответе клетки на оксидативный стресс, вероятно, подвергался отбору не только у якутского скота и коренного населения Сибири, но также у местных якутских лошадей (Librado et al., 2015). Хронический холодовой стресс увеличивал экспрессию мРНК гена SOCS3 в гипоталамусе и периферических мононуклеарных клетках крови у крыс и хорьков (Reynés et al., 2017). Три гена (PFKM, ADAM17 и SIRPA), подвергавшихся селекции у якутского скота, оказались ассоциированы с устойчивостью к заболеваниям (Weldenegodguad et al., 2019). Так, белок ADAM17 регулирует восприятие болевых стимулов, в том числе и холода (Quarta et al., 2019).

Сравнительный анализ данных полногеномного секвенирования китайской турано-монгольской породы Yan-bian и африканской тауринной породы N’Dama показал наличие у животных обеих пород следов отбора в гене CD36 (Shen et al., 2020). Белок CD36 играет важную роль в мембранном транспорте жирных кислот в сердце, скелетной мускулатуре и жировой ткани (Glatz et al., 2010). Экспрессия CD36 увеличивается при холодовом воздействии, что повышает поглощение бурой жировой тканью триглицерид-богатых липопротеинов (Bartelt et al., 2011). У голодающих мышей, нокаутных по гену CD36, вскоре после воздействия холода температура тела резко снижалась, причем эта гипотермия сопровождалась заметным снижением как уровня глюкозы в крови, так и запасов триацилглицеринов в бурой жировой ткани и гликогена в скелетной мускулатуре (Putri et al., 2015). Известно, что у животных породы Yanbian экспрессия гена CD36 положительно коррелирует с содержанием внутримышечного жира (мраморностью) (Jeong et al., 2012). По-видимому, именно обширные запасы жира способствуют резистентности к холоду у животных. Эти же авторы выявили достоверный сигнал в гене FGF5 по данным поиска районов отбора у породы Yanbian (Shen et al., 2020). Белок FGF5 регулирует рост волосяного фолликула и длину волоса у кошки, собаки и человека (Higgins et al., 2014). Порода Yanbian характеризуется длинным и густым волосяным покровом, который помогает ей адаптироваться к зимним температурам до –37 °С.

**Гены иммунного ответаM**

Одна из первых работ по поиску следов селекции у двух турано-монгольских пород – якутской и калмыцкой – была выполнена путем генотипирования ОНП маркеров на чипе Illumina BovineSNP50 (Iso-Touru et al., 2016). У калмыцкой породы и четырех других пород авторы нашли следы селекции в районе 4 116 037–4 616 037 п.н. на хромосоме 16, который содержит шесть генов иммунной системы (IL10, IL19, IL20, PIGR, FCAMR и IL24). У тураномонгольской якутской и тауриновой серой украинской породы следы селекции были выявлены на хромосоме 21 в районе 33 802 673–35 302 673 п.н., который содержит четыре гена иммунной системы (CSK, GZMB, PML и SEMA7A). 

Секвенирование транскриптома крови показало, что у животных якутской породы по сравнению с голштинской повышена экспрессия 89 генов (Pokharel et al., 2019). К числу семейств, в которых повышена экспрессия двух и более генов, относятся хемокины (CCL4, CCL5), углеводные сульфотрансферазы (CHST1, CHST12), хемокиновые рецепторы (CX3CR1, CXCR6), блокирующие рост специфические белки (GAS6, GAS7), гранзимы (GZMB, GZMM, GZMH), белки, связывающие инсулиноподобный фактор роста (IGFBP4, IGFBP7), а также рецепторы естественной цитотоксичности (NCR1, NCR3). Так, у якутского скота авторы выявили повышение экспрессии четырех транскриптов гранзимов и перфорина. Гранзимы являются сериновыми протеазами, которые используются цитотоксическими лимфоцитами для уничтожения злокачественных и зараженных вирусом клеток. Гранзимы транспортируются в цитоплазму клетки-мишени перфорином 1 (PRF1), после чего они расщепляют специфические белки и запускают апоптоз (Johnson et al., 2003). Полученные данные свидетельствуют о наличии очень сильной опосредованной гранзимами иммунной реакции у якутского скота.

Полногеномное секвенирование представителей породы ханву позволило выявить протяженный район гомозиготности вблизи гена RCN2 (LeeT. et al., 2013). По мнению авторов, именно отбор по гену RCN2 привел к формированию у ханву устойчивости к вирусу папилломы КРС. Другие авторы использовали данные полногеномного секвенирования для поиска породоспецифических генов у ханву путем анализа прочтений, которые не выравнивались на референсный геном (Caetano-Anolles et al., 2018). Оказалось, что значительное число белковых доменов этих генов ассоциировано с функцией иммуноглобулинов и они потенциально могут взаимодействовать с доменами других белков иммунной системы.

Генотипирование животных породы ханву примерно по 680 тысячам ОНП позволило найти следы отбора в локусах, содержавших целый ряд генов иммунной системы, например CD14, CD28, RFX5, TNFAIP8L2, TMEM173, IL17D, TGFB1 и NFKBIA (Porto-Neto et al., 2014). Белок, кодируемый геном CD14, является поверхностным антигеном, который экспрессируется преимущественно на мо-ноцитах/макрофагах и участвует в формировании врожденного иммунитета на бактериальные липополисахариды (Tsukamoto et al., 2018). Рецептор CD28 связывается с токсинами стафилококков и стрептококков и запускает выделение цитокинов и Т-клеточный иммунный ответ (Kaempfer et al., 2013). Интерлейкин-17D (IL17D) стимулирует инфильтрацию нейтрофилов, естественных киллеров и моноцитов в ответ на инфекцию цитомегаловируса у мышей (Seelige et al., 2018). Транскрипционный фактор RFX5 опосредует экспрессию генов MHC-II и, таким образом, играет значительную роль в адаптивном иммунном ответе (Garvie et al., 2007). Белок TNFAIP8L2 считается репрессором врожденного и адаптивного иммунитета и участвует в поддержании иммунного гомеостаза (Niture et al., 2019). Белок STING, кодируемый геном TMEM173, входит в состав белкового комплекса, который распознает нуклеиновые кислоты вирусов и бактерий в цитозоле и активирует транскрипцию интерферонов первого типа (Motwani et al., 2019). Экспрессия мРНК NFKBIA в линии клеток почки свиньи изменялась при инфицировании вирусом ящура (Zhang T. et al., 2018).

Анализ регионов с вариацией по числу копий ДНК (CNV) по данным полногеномного секвенирования животных, представляющих шесть аборигенных китайских пород скота (Leiqiong, Nanyang, Luxi, Yunnan, Qinchuan, Yanbian) и две интернациональные специализированные мясные породы (вагу, красный ангус), позволил выявить 11 486 CNV регионов, покрывающих 52.04 млн п. н. (1.96 %) от референсного генома (Mei et al., 2019). У китайского скота авторами были идентифицированы многочисленные локализованные в CNV регионах гены, которые связаны с иммунным ответом. Так, ген BOLA-DQA2 может быть критическим фактором в резистентности к маститу у молочного скота (Hou et al., 2012). CNV в этом гене ассоциирована с иммунным ответом у яка (Zhang X. et al., 2016). Сообщалось, что гены AUH, CXCL14, IL1RL2 и EPB42 влияют на толерантность к паразитам у различных пород скота (Mustafa et al., 2018). Ряд генов, в том числе A2M, BLA-DQB, HSPA9, IFNA16, IL15, IL2, PROCR, SP3, VAMP7 и TLR4, связаны, по данным многочисленных исследований, с иммунным ответом (Mei et al., 2016; Randhawa et al., 2016). Несколько генов иммунной системы активируются в ответ на внешнее воздействие, например EGLN2 (Wu D.D. et al., 2018), SOX5 (Liu, Bickhart, 2012), CD59 (Chan et al., 2010), GPR50, TAS2R16 (Gautier et al., 2016), NFIB (Zhao et al., 2017), а также FZD10, KIF2B и ROBO1 (Ai et al., 2015).

Сравнение путем секвенирования РНК дифференциально экспрессирующихся генов в печени и селезенке показало, что экспрессия некоторых генов, связанных с иммунной функцией (C1QB, CD55, MASP2, C4BPA, MAVS, NOD2 и CD46), была повышена у животных породы Yunnan humped по сравнению с голштинами, в то время как экспрессия других генов (C2, SERPING1, SERPINE1, TIRAP, TLR2 и TLR6) была понижена (ChenY. et al., 2019). Ген C1QB кодирует В-цепь компонента комплемента 1q, участвуя в формировании врожденного иммунитета, а также считается одним из хабов реакции организма на инфицирование Mycobacterium tuberculosis (Sambarey et al., 2017). Ген CD46 кодирует белок, который является компонентом системы комплемента и может служить в качестве рецептора для вируса кори, герпес-вируса человека 6-го типа и бактерии Neisseria (Yamamoto et al., 2013). Другой белок комплемента, CD55, ассоциирован с малярией и аутоиммунными заболеваниями (Dho et al., 2018). Компонент комплемента С2 участвует в очищении тканей от апоптотических клеток, и генетические варианты этого гена ассоциированы с красной волчанкой (Chen H.H. et al., 2015). Ген MASP2 кодирует белок, который относится к семейству сериновых пептидаз S1. В исследовании (Kasanmoentalib et al., 2017) мыши с нокаутом по этому гену чаще гибли, по сравнению с контролем, при пневмококковом менингите. Белок C4BPA входит в состав мультимерного белка C4BP, контролирующего активацию комплемента по классическому пути. C4BP связывается некоторыми патогенами, в частности Streptococcus pyogenes, что обеспечивает этим бактериям выживание в организме хозяина (Ermert, Blom, 2016). Белок MAVS необходим для активации транскрипционных факторов, которые регулируют экспрессию бета-интерферона и, таким образом, запускают реакции противовирусного иммунитета (Belgnaoui et al., 2011). Ген NOD2 экспрессируется преимущественно в лейкоцитах. Его белковый продукт участвует в иммунном ответе на бактериальные липополисахариды путем распознавания мурамилдипептида и активации белка NFkB (Kuss-Duerkop, Keestra-Gounder, 2020). У человека ОНП маркеры в гене SERPINE1 ассоциированы с повы-шенной смертностью от сепсиса (Shi et al., 2015). Толлподобный рецептор TLR6 образует гетеродимерный комплекс с TLR2, который распознаёт целый ряд патоген-связанных молекулярных структур. ОНП маркеры в генах TLR2 и TLR6 ассоциированы с чувствительностью КРС к Mycobacterium avium spp. paratuberculosis, а также к туберкулезу и филяриозу у человека (Mukherjee et al., 2019). Генотипирование на чипах высокой плотности гибридов вагу с породой Fuzhou позволило идентифициро-вать следы отбора в ряде генов (TLR9, TAFA1, GPX8, IL5, PRDM9 и GRIK2), ассоциированных с иммунной функцией (Wang Z. et al., 2019). Внутриклеточный толл-подобный рецептор TLR9 обычно распознает патогены, проникающие внутрь клетки (Mukherjee et al., 2019). ОНП маркеры в гене TLR9 у человека ассоциированы с иммунным ответом при туберкулезе (Bharti et al., 2014). Ген TAFA1 кодирует небольшой, сходный с хемокинами белок, который экспрессируется преимущественно в определенных областях мозга и функционирует в качестве регулятора иммунных и нервных клеток (нейрокина) (Tom Tang et al., 2004). Цитокин IL5 является фактором роста и дифференцировки В-клеток и эозинофилов (Takatsu, 2011).

**Гены адаптации к высокогорью**

Как полагают, як обитает на Тибетском плато на протяжении миллионов лет и за это время приобрел многочисленные адаптации к условиям высокогорья, например увеличенный размер легких и сердца. В отличие от яка, домашний тауринный скот появился на Тибетском плато вместе с людьми всего несколько тысяч лет назад. Быстрой адаптации тибетского скота к условиям высокогорья, вероятно, способствовала интрогрессия генов сигнального пути ответа на гипоксию (например, EGLN1, EGLN2 и HIF3a) (Wu D.D. et al., 2018). Эти же авторы показали, что тибетский скот, имеющий гаплотип EGLN1 от яка, тоже имеет сниженную концентрацию гемоглобина и число эритроцитов в крови, что расценивается как адаптивный признак.

Другая группа исследователей на основании данных полногеномного секвенирования установила, что в среднем 1.22 % генома тибетского скота произошли в результате интрогрессии от яка примерно две тысячи лет назад (Chen N. et al., 2018a). Анализ списка интродуцированных генов выявил достоверное обогащение тремя терминами генной онтологии: сенсорное восприятие запаха (GO:0007608), трансмембранный транспорт L-орнитина (GO:1903352), а также процессинг антигена и презентация пептидных или полисахаридных антигенов с помощью белков главного комплекса гистосовместимости класса II (GO:0002504). Поиск по базе данных KEGG показал, что наибольшая группа интродуцированных генов была вовлечена в реакцию «трансплантат против хозяина». В геноме тибетского скота в районах интрогрессии было найдено несколько генов, которые могли участвовать в адаптации к гипоксии. К ним относятся гены метаболического пути индуцируемого гипоксией фактора – COPS5, IL1A, IL1B, MMP3 и EGLN1, которые неоднократно были идентифицированы в качестве мишеней для отбора на адаптацию к высокогорью у жителей Анд, Тибета и у яка (Bigham et al., 2010; Qiu et al., 2012). Два гена, RYR2 и SDHD, участвуют в регуляции гомеостаза кальция, который опосредует реакцию на гипоксию (Wang M.S. et al., 2015).

Сравнительный анализ районов CNV у высокогорного тибетского скота и равнинного монгольского (Menggu) по данным полногеномного генотипирования на биочипе Illumina BovineHD Genotyping BeadChip позволил идентифицировать три потенциальных гена-кандидата (LETM1, TXNRD2 и STUB1) адаптации к гипоксии (Zhang Y. et al., 2020). Ген LETM1 кодирует белок, встроенный во внутреннюю мембрану митохондрий, который играет существенную роль в поддержании нормальной морфологии этих органелл и жизнеспособности клеток (Li Y. et al., 2019). Опосредованная аденовирусом сверхэкспрессия гена LETM1 может приводить к снижению выработки АТФ, потребления кислорода и массы митохондрий, а также к некротической гибели клеток HeLa (Piao et al., 2009). В геноме тибетского скота было выявлено пониженное число копий гена LETM1, что может способствовать адаптации этих животных к гипоксии путем сохранения нормальной морфологии и жизнедеятельности митохондрий. Ген TXNRD2 кодирует митохондриальную тиоредоксин-редуктазу типа 2. У мышей со специфическим нокаутом этого гена в сердце наблюдаются дегенерация митохондрий и стабилизация фактора HIF-1aльфа (Kiermayer et al., 2015). Белок STUB1 представляет собой E3 убиквитинлигазу и играет существенную роль в убиквитинировании и деградации фактора HIF-1aльфа (Ferreira et al., 2013).

## Заключение

Проведенный нами анализ литературы позволяет сделать следующие выводы:

- турано-монгольским породам свойственна высокая адаптация к экстремальным климатическим условиям и паразитам, обусловленная селекцией в районах генов иммунного ответа и терморегуляции;
- несмотря на активное смешение с коммерческими породами, древний предковый генетический компонент турано-монгольского скота все еще присутствует во многих разводимых в Азии породах КРС, а якутский скот остался, по-видимому, единственным чистокровным носителем этой компоненты;
- имеющиеся в настоящее время данные литературы не позволяют отдать предпочтение одному из двух возможных сценариев происхождения турано-монгольских пород – в результате доместикации дикого тура на территории Восточной Азии или вследствие миграции тауринной протопопуляции из Ближнего Востока;
- в результате высокопроизводительного генотипирования и секвенирования геномов и транскриптомов представителей турано-монгольских пород в последнее время были найдены перспективные гены-кандидаты, участвующие в адаптации к факторам внешней среды.

Дальнейшие исследования в этой области должны быть направлены на: 1) накопление генетических и палеогенетических данных, которые позволят сделать окончательный вывод о происхождении турано-монгольской группы пород; 2) создание референсного турано-монгольско-го генома и его независимой аннотации для детального сравнения турано-монгольских пород, поскольку значительные отличия от существующего референсного генома (герефорда) могут приводить к невыявлению генетических различий, характерных только для турано-монгольских пород в сильно дивергированных участках их геномов; 3) определение роли высокочастотных замен, характерных для турано-монгольских пород, и их введение в селекционно-племенную работу коммерческих пород путем генного редактирования.

## Conflict of interest

The authors declare no conflict of interest.
